# Environmental Performance Evaluation of Key Polluting Industries in China—Taking the Power Industry as an Example

**DOI:** 10.3390/ijerph19127295

**Published:** 2022-06-14

**Authors:** Zuoming Liu, Changbo Qiu, Min Sun, Dongmin Zhang

**Affiliations:** 1 School of Business and Management, Jilin University, Qianjin Street 2699, Changchun 130012, China; lzmhero_@126.com (Z.L.); qiucb@jlu.edu.cn (C.Q.); 2 School of Statistics, Jilin University of Finance and Economics, Jingyue Street 3699, Changchun 130117, China; 18234175310@163.com

**Keywords:** key polluting industries, power industry, environmental performance, optimization path, super-efficient MinDS model

## Abstract

This paper analyzes the environmental performance, spatial and temporal characteristics, and optimization paths of key polluting industries, represented here by the power industry, using the super-efficient MinDS model. The study shows that the environmental performance as a whole presents the characteristics of an inverted U-shaped and then a U-shaped trend; each region presents an asymmetric state of convergent development followed by differentiated development, with 2014 as the structural change point; the development trend of environmental performance in each region is divided into three categories (rising, falling, and stable) and four types of spatial clustering (ultra-high, high, medium, and low levels); and input–output indicators of environmental performance in China and across regions have varying degrees of redundancy, with labor input redundancy being the greatest, followed by capital input, technology input, and pollution emissions. On this basis, we propose to improve the monitoring and inspection mechanism of the implementation process of pollution control in key polluting industries and to improve the level of environmental performance of key polluting industries by optimizing the combination of labor, capital, and technology input factors in each region according to local conditions and adopting differentiated strategies. The main contributions of this paper are threefold: first, we incorporate technological inputs into the environmental performance evaluation index system of the electric power industry, which can better reflect the real inputs of the electric power industry and measure the results more accurately; second, we adopt the MinDS model for measuring the environmental performance level, which can quantitatively analyze the gap between each indicator and the optimal level; and third, we propose a redundancy index, which can be used to compare the redundancy of each indicator and then judge the main efficiency levels of the different factors.

## 1. Introduction

At present, China’s economy is in the transition stage from stable growth to high-quality development, and the coordinated development of the economy and the environment is an inevitable requirement for high-quality development. However, the problem of carbon emissions in China is still serious, and environmental problems have seriously restricted the high-quality development of China’s economy. According to relevant statistics, industries with large energy consumption, such as electricity, transportation, construction, and industry, are the main sources of carbon emissions in China, and the sum of their carbon emissions exceeds 74% of the total national carbon emissions [1,2,3,4]. In 2016, the National Development and Reform Commission and the National Energy Administration jointly issued the Energy Production and Consumption Revolution Strategy (2016–2030), which proposed actively controlling carbon emissions and implementing supply-side energy. In the United Nations General Assembly in 2020, China made a solemn commitment to the international community that its CO_2_ emissions would peak in 2030 and that it would strive to achieve carbon neutrality by 2060 [5,6,7]. In fact, in the carbon emission structure of the energy sector, carbon emissions from the power industry are predominant [8,9,10]. According to the latest data published by the International Energy Agency (IEA), global energy-related CO_2_ emissions reached a record high of 33.1 billion tons in 2018, an increase of 1.70% over the previous year and the highest growth rate since 2013. Among them, global CO_2_ emissions from the power sector reached 13 billion tons, accounting for 38% of total energy-related CO_2_ emissions. China is the world’s largest producer of electricity and the country with the largest increase, accounting for 26.70% of the world’s total power generation capacity. According to the data provided by the 13th Five-Year Plan for Electricity Development (2016–2020), the carbon emissions of coal-fired units in China are as high as 890 g/kWh, while those of the United States and Japan are 433 g/kWh and 544 g/kWh, respectively. Thus, it can be seen that there is some room for decarbonization in China’s electric power and that it is the “front-runner” in terms of controlling carbon emission reduction [11,12,13,14]. Although the power industry is actively committed to the development of new energy sources, it still uses coal as the main raw material, which is highly polluting and highly emitting; therefore, the power industry has the most difficult task and the greatest responsibility when it comes to reducing carbon emissions, and as such it plays a major role in the realization of the dual carbon goal. Therefore, this paper takes the electric power industry as an example; constructs an environmental performance evaluation index system; evaluates the environmental performance levels of key polluting industries in China; explores the spatial and temporal characteristics of the environmental performance level of each province, autonomous region, and municipality under the direct control the central government in China; and further quantitatively analyzes the gap between the current environmental performance level and the optimal level, and on this basis proposes a path to improve the environmental performance level of key polluting industries in China. The research contained in this paper provides important theoretical value and practical guidance for improving the environmental efficiency level of key polluting industries in China and promoting sustainable economic development and deep green development in China.

## 2. Literature Review

Environmental performance, also known as environmental efficiency or eco-efficiency, was first introduced by Schaltegger and Sturm in 1990 [15]. Environmental performance was defined as “the ratio of the value added of an economy to its environmental impact”; in 1998, The Organization for Economic Cooperation and Development (OECD) expanded the meaning of environmental performance. The OECD considers environmental performance as the efficiency of ecological resources to meet human needs and as an input–output relationship. Output refers to the value of goods and services provided by economic activities, and input refers to the resources consumed by economic activities and the resulting environmental load. This is the most commonly applied definition.

In recent years, with the increase in public concern about the environment and the successive introduction of government policies, people have been paying more and more attention to the environmental effects of enterprise production activities. Many scholars have started to pay attention to the environmental governance of heavily polluting industries [16,17,18]. Two ideas were mainly used to carry out these kinds of studies. One is to select representative industries, such as electricity, cement, and steel, for the study, and the other is to select the most polluting power industry as a representative for the study. The measures of environmental performance are also mostly represented simply by the impact of production activities on the environment. The two methods of research are relatively similar, but the second one is more common. Therefore, in this paper, we only review the literature on environmental performance in the power industry.

Looking at the previous literature on the selection of environmental performance indicators in the power industry, it is difficult to find studies with identical indicators. Peng selected sulfur dioxide emissions [19], nitrogen oxide emissions, and soot emissions from the thermal power industry as input indicators and gross regional product as output indicators based on the definition of eco-efficiency. The dynamic trend of the environmental performance of China’s power industry during the period from 2006 to 2009 was measured. The study concluded that the environmental performance of the electric power industry in China as a whole and in all regions has improved significantly, but the degree of improvement varies greatly between provinces, with the greatest improvement in the environmental performance of the electric power industry being found in northern China. Most scholars selected the number of employed persons, power generation equipment capacity, and fuel consumption as input indicators, power generation as desired output indicators, and environmental pollution emissions as non-desired output indicators to measure environmental performance [20,21,22,23,24]. The research shows that the ecological efficiency, technical efficiency, and energy efficiency of China’s power industry have been significantly improved, and there are large regional differences; Qu et al. (2012) and Zhu (2015) added plant electricity consumption as an input indicator to these indicators to measure the technical input level of electricity [25,26]. The research shows that there are large spatial and industrial differences in the ecological efficiency of the power industry, and the production efficiency of thermal power enterprises is higher than that of hydropower enterprises. Wang and Zhu [27] and Wang et al. [28] added equipment utilization hours to the input indicators. The research shows that the coordination degree of the energy and environmental development of the power industry in the eastern region is higher than that in the central and western regions; Fan and Yuan [29] added transmission line length as an input indicator of the grid link and increased the proportion of clean energy generation as the expected output indicator of grid link. The research shows that the economic and technical efficiency of China’s power industry is significantly higher than the environmental and social technical efficiency. In general, for input indicators, most of the literature considers asset inputs, labor inputs, and fuel consumption inputs; for output indicators, most of the literature takes power generation as the desired output and pollution emissions as the non-desired output.

For the measurement methods of environmental performance, researchers have mainly used life cycle analysis (LCA), stochastic frontier analysis (SFA), and data envelopment analysis (DEA) methods. The LCA method mainly focuses on a specific product and measures the environmental efficiency of the product throughout its life cycle, which makes it difficult to evaluate the environmental efficiency of enterprises or industries. The SFA method is a parametric method that requires the determination of efficiency when setting a specific production function and using statistical methods to estimate the parameters, which may result in large errors. Fare and Lovell [30] first proposed the environmental efficiency evaluation method based on DEA. DEA is a non-parametric method that can overcome the shortcomings and errors caused by the subjective setting of functions and weights by parametric methods because it does not utilize any functional form of assumptions [31,32,33,34]. Additionally, it can effectively evaluate the efficiency of decision units with multiple inputs and multiple outputs; so, DEA is the most widely used environmental efficiency evaluation method at present [35,36,37,38,39,40]. For studies on the environmental performance of the power industry, there is little literature dedicated to the environmental performance of the power industry; most of the literature incorporates environmental constraints and conducts studies in terms of the production efficiency, operational efficiency, and technical efficiency of the power industry. Korhonen and Luptacik [41] evaluated the eco-efficiency of 24 power plants in European countries using an extended DEA model. Bai and Song [42] and Sueyoshi et al. [43] used a three-stage DEA model. The research shows that the ecological efficiency, technical efficiency, and energy efficiency of China’s power industry have been significantly improved and that there are large regional differences. Arabi et al. [44] and Munisamy and Arabi [45] similarly used the SBM model to measure the Malmquist–Luenberger productivity of the power industry under ecological and environmental constraints. Wang and Yang [46] used the NSBM model to study the environmental technical efficiency and total factor productivity of the power industry in China. Halkos and Polemis [47] estimated the efficiency of the U.S. power generation industry using a window DEA model.

Previous studies on the construction and measurement of environmental performance index systems in the power industry have provided us with many insights, but there are also certain problems. First, there is no uniform standard for the selection of environmental performance indicators. For the input indicators, previous scholars have considered three aspects: asset input, labor input, and fuel consumption input; in addition, there are a large number of technical inputs in the power industry, such as power plant consumption rate, line loss rate, and transmission line length [48,49,50,51,52]. Although a few scholars consider these indicators, they are rather one-sided and do not include all aspects of power generation, transmission, and distribution. Therefore, we add three technical input indicators, such as power plant consumption rate, line loss rate, and transmission line length, and downscale these three indicators using the entropy method with objective weighting to reflect the technical input of the power industry in a comprehensive manner. Second, regarding the measurement methods of environmental performance in the electric power industry, previous scholars mainly used the traditional DEA model, super-efficient DEA model, three-stage DEA model, SBM model, and NSBM model to measure the environmental performance of the electric power industry, but all of them have certain defects; namely, the projection point of the inefficient decision unit on the frontier surface is always the farthest point from the evaluated DMU, resulting in the improvement path of inefficient DMUs being more difficult to implement. Moreover, previous scholars have not quantified the input redundancy or output deficiency of inefficient DMUs; so, the input and output indicators affecting inefficient DMUs cannot be found at the root [53,54,55,56,57]. Additionally, the nearest distance to the strong efficient frontier (MinDS) model uses the shortest distance from the frontier surface to evaluate the efficiency value of the DMU, so that the environmental performance takes the shortest path from inefficient to efficient, making the proposed improvement plan easier to achieve and more conducive to the formulation of policy recommendations. Based on the above considerations, we intend to add technical input indicators to the traditional index system of environmental performance in the power industry, select the super-efficient MinDS model to evaluate and analyze the environmental performance of China’s power industry, and the spatial and temporal differences, and explore the improvement paths of environmental performance in the power industry in inefficient provinces.

## 3. Methodological Description and Selection of Indicators

### 3.1. Methodological Description

The super-efficient MinDS model combines the super-efficiency model and the minimum distance to strong efficient frontier (MinDS) model. Andersen and Petersen [58] first proposed the super-efficiency model in 1993, which yielded practical DMU efficiency values greater than 1; so, this model can further distinguish the efficiency of the effective DMU. Then, the Tobit regression model, which deals with truncated data, is also not required to analyze the factors influencing efficiency. The core idea of the super-efficiency model is to remove the evaluated DMU from the reference set and derive the efficiency value of the evaluated DMU by referring to the frontier composed of other DMUs. Aparicio [59] first proposed the MinDS model in 2007, which is an improvement on the frontier furthest distance model (SBM) and uses the shortest distance from the frontier surface to rate the efficiency value of the DMU. The MinDS model uses the shortest distance from the frontier plane to evaluate the efficiency values of the DMUs. This model applies a mixed-integer linear programming approach by adding constraints to restrict all evaluated DMUs to the same hyperplane and then determines all efficient DMUs by the SBM model and solves the planning model with an efficient subset as its reference set [60,61,62,63]. Compared with the traditional DEA model and SBM model, the MinDS model has the following advantages: (1) it addresses the problem of slackness of variables not considered in traditional DEA models; and (2) it overcomes the problem that the objective function of the SBM model minimizes the efficiency value. Based on the above theory, we establish the super-efficient MinDS model in two steps.

The first step is to establish the super efficiency SBM model and determine the effective DMU set, which is composed of a DMU with an efficiency level greater than 1; that is, $E=\left\{j|\rho_{j}^{\prime }\geq 1\right\}$, where $\rho^{\prime }$ represents the efficiency value of the evaluated DMU and j represents the first DMU. The objective function and constraints of solving the effective DMU set are as follows:

(1) $$Min\rho^{\prime }=\frac{1-\frac{1}{m}\sum_{i=1}^{m}s_{i}^{-}/x_{ik}}{1+\frac{1}{q}\sum_{r=1}^{q}s_{r}^{+}/y_{rk}}$$

(1a) $$s.t.\;\sum_{j=1,j\neq k}^{n}x_{ij}\lambda_{j}+s_{i}{}^{-}=x_{ik},i=1,2,\cdots ,m$$

(1b) $$\sum_{j=1,j\neq k}^{n}y_{rj}\lambda_{j}-s_{r}{}^{+}=y_{rk},r=1,2,\cdots ,q$$

(1c) $$\sum_{j=1,j\neq k}^{n}\lambda_{j}=1,j=1,2,\cdots ,n(j\neq k)$$

In the above equation, (1) represents the objective function, and (1a)–(1c) represent constraints. $s_{i}^{-}$ and $s_{r}^{+}$ represent the relaxation variables of input indicators and output indicators, respectively; $x_{ij}$ denotes the *i*-th input indicator of the *k*-th DMU; $y_{rk}$ denotes the *r*-th output indicator of the *k*-th DMU; m denotes the number of input indicators; q denotes the number of output indicators; $x_{ij}$ denotes the *i*-th input indicator of the *j*-th DMU other than k; $y_{rj}$ denotes the *r*-th output indicator of the *j*-th DMU other than k; and $\lambda_{j}$ is the weight vector. When $\rho^{\prime }\geq 1$, the evaluated DMU is relatively effective. When $\rho^{\prime }\leq 1$, the evaluated DMU is relatively ineffective, so the input–output indicators need to be improved.

The second step is to establish a mixed integer linear programming model under the constraints of the effective DMU set, calculate the super efficiency minds value, and solve the objective function and constraints of the super efficiency minds value model as follows:

(2) $$\max\rho =\frac{\frac{1}{m}\sum_{i=1}^{m}\left(1-s_{i}^{-}/x_{ik}\right)}{\frac{1}{q}\sum_{r=1}^{q}\left(1+s_{r}^{+}/y_{rk}\right)}$$

(2a) $$s.t.\;\sum_{j\in E,j\neq k}x_{ij}\lambda_{j}+s_{i}^{-}=x_{ik},\;i=1,2,\ldots ,m$$

(2b) $$\sum_{j\in E,j\neq k}y_{rj}\lambda_{j}-s_{r}^{+}=y_{rk},\;r=1,2,\ldots ,q$$

(2c) $$s_{i}^{-}⩾0,\;i=1,2,\ldots ,m$$

(2d) $$s_{r}^{+}⩾0,\;r=1,2,\ldots ,q$$

(2e) $$\lambda_{j}⩾0,j\in E,j\neq k$$

(2f) $$-\sum_{i=1}^{m}v_{i}x_{ij}+\sum_{r=1}^{q}\mu_{r}y_{rj}+d_{j}=0,j\in E,j\neq k$$

(2g) $$v_{i}\geq 1,i=1,2,\cdots ,m$$

(2h) $$\mu_{r}\geq 1,r=1,2,\cdots ,q$$

(2i) $$d_{j}\leq Mb_{j},j\in E,j\neq k$$

(2j) $$\lambda_{j}\leq M(1-b_{j}),j\in E,j\neq k$$

(2k) $$b_{j}\in \left\{0,1\right\},j\in E,j\neq k$$

(2l) $$d_{j}\geq 0,j\in E,j\neq k$$

It can be seen from Equation (2) that the minds model is composed of three parts: objective function (1), constraint b, constraint c, and constraint d. In the above equation, the meanings of $s_{i}^{-}$$s_{r}^{+}$, $x_{ik}$, $y_{rk}$, m, q, $x_{ij}$, $y_{rj}$, i, j, k, r, n, and λ are the same as those mentioned above. M is a sufficiently large positive number and $v_{i}$ and $\mu_{r}$ represent the weight of the input index and the output index, respectively. It can be seen from the above equation that if the evaluated DMU wants to achieve the optimal efficiency, the necessary and sufficient condition is that all relaxation variables are 0. Equations (2a) and (2b) have the same meaning as Equations (1a) and (1b), except that they are limited to the effective DMU set. The common goal of constraints c and d is to ensure that the reference posts are located in the same hyperplane. Equation (2f) is a mixed integer linear constraint. The feasible solution is the hyperplane where all reference benchmarks are located. It can make the programming model automatically take the effective subset as its reference set, avoiding the process of testing all subsets. Equations (2i)–(2l) are the constraints.

### 3.2. The Construction of the Index System

We comprehensively consider the ISO14031 environmental performance evaluation system and WBCSD eco-efficiency index system to select the input–output indicators, and the selection of the index system follows the principles of scientificity, validity, measurability, comparability, and systematization. Since the electric power industry is mainly divided into three sectors—the power generation sector, the transmission sector, and the distribution and sales sector—we should include each of them as much as possible in the selection of indicators in order to reflect the environmental performance of the whole electric power industry comprehensively. The basis for the selection of the input and output indicators of the power industry is shown below, and we summarize all the input and output indicators into a table to constitute the environmental performance indicator system of the power industry, as shown in Table 1.

Capital input: This reflects the resource input of electric power enterprises. Since it is difficult to measure the input capital of the power industry, this paper selects the installed capacity indicator, i.e., the overall power of the enterprise’s generating units, to reflect the scale of production capacity input in each region.

Labor input: We usually use three methods to measure labor input in the power generation industry: labor time, labor income, and labor headcount. However, due to the residual administrative monopoly in China’s power generation industry, the labor income of employees in the power industry is not a reflection of the value of labor under market conditions. As we do not count the labor time data, we finally chose the number of laborers to represent the labor input. Since it is difficult to obtain the number of employees in the electric power industry in each province, city, and autonomous region directly, we used the number of employees in the heat and power production and supply industry, which is highly related to the electric power industry, to represent the number of employees.

Fuel input: This refers to the fuel consumed by power industry enterprises in the process of power generation, which can reflect the degree of fuel utilization. At present, China mainly focuses on thermal power generation, and the energy input required for thermal power generation is mainly coal and fossil energy such as oil, natural gas, and coke. In order to make these energy inputs comparable with each other, we need to convert these energy sources into standard coal consumption uniformly, and finally we express the energy inputs in terms of standard coal consumption for power generation.

Technical input: We measure the technical input of the region through power consumption rate in the power plant, line loss rate, and transmission line length. The power consumption rate in the power plant refers to the percentage of electrical energy consumed by the power plant in producing electrical energy of the total amount of electrical energy generated, which reflects the rate of power consumption in the generation chain. The line loss rate refers to the percentage of electrical energy lost in the transmission and distribution process to the electrical energy supplied by the power network and measures the economy of power system operation. The length of transmission lines is directly proportional to the voltage, so there is also a particular influence on line losses.

Electricity generation: This refers to the amount of electrical energy produced by a power plant, which is the value of the main product or service of the power industry.

Gross regional product: Electric energy, as an essential secondary energy source, provides power for all fields of social production, and electricity consumption shows a strong positive correlation with economic growth; therefore, it is feasible to measure the economic output of the regional power industry using the regional GDP.

Environmental pollution: According to the principle of conservation of matter, fuel consumption causes environmental pollution. The pollutants produced by the power industry are mainly CO_2_ emissions, SO_2_ emissions, and nitrogen oxides; so, we chose these three environmental pollution indicators.

### 3.3. Data Sources and Processing

Since data from Tibet, Hong Kong, Macau, and Taiwan are difficult to obtain, this paper analyzes data from the remaining 30 provinces, autonomous regions, and municipalities under the direct control of the central government in China. According to the availability of data, we selected the data of the last ten years, i.e., 2010–2019, for the study. The data on labor force and gross regional product were obtained from the China Statistical Yearbook and all other data were obtained from the China Electricity Yearbook. For the few missing values, interpolation and extrapolation methods were used to obtain them. In order to eliminate the influence of price factors, the data of gross regional product were calculated in constant prices with 2010 as the base period.

We obtained data on fuel coal consumption and nitrogen oxide emissions in the thermal power industry from the Annual Report of China Environmental Statistics. Currently, the Chinese Yearbook does not contain data on pollution emissions in the power industry by region for all years. Some literature directly uses industrial emissions instead of power industry emissions, which is likely to affect the accuracy of the results. To make up for this deficiency, we based this analysis on the results of the first national pollution source survey of the State Council in 2008 and the emission coefficient of major pollutants in the thermal power industry calculated by the State Grid Group of China and the State Grid Institute of Environmental Protection of China. The determination methods of pollutant emissions of thermal power plants include the measurement method, material balance algorithm, element balance method, production and emission coefficient method, etc. The production and discharge coefficient method has the advantages of simplicity, clarity, and easy operation. Therefore, this method is the most general and effective technical means of determination at present. We estimated the carbon dioxide emissions based on the changes in three driving factors, namely, power generation, coal consumption for power supply, and carbon emission intensity, as shown in Equation (3). We estimated the sulfur dioxide emissions based on the power generation, the sulfur content of coal combustion, the emission factors of different combustion equipment, and the efficiency of pollutant treatment, as shown in Equation (4).

(3) $$CO_{2}=Q\times E\times \delta_{i}$$

(4) $$SO_{2}=1.6\times Q\times \omega \times (1-\eta )$$

In Equations (3) and (4), $CO_{2}$ is the carbon emissions, Q is the electricity generation, E is the coal consumption for the electricity supply, δ is the carbon emission factor for standard coal, $SO_{2}$ is the sulfur dioxide emission, Q is the power generation, ω is the coal consumption per unit of power generation, and η is the desulfurization efficiency of the desulfurization facility.

Considering that the DEA model should make the input–output indicators as concise as possible when it is applied in practice, we use the entropy value method to downscale the three indicators of technological inputs and the three indicators of environmental pollution emissions and synthesize a total input technology indicator and a total environmental pollution emission indicator, respectively. The descriptive statistical results of the input–output index system of environmental performance of the power industry are shown in Table 2.

## 4. Analysis of Empirical Results

### 4.1. Measurement of Environmental Performance in China’s Power Industry

#### 4.1.1. Time-Series Characteristics of the Power Industry

The software used in this study was MaxDEA 8 Ultra. The first version of MaxDEA was developed by ChengGang in 2009, and then ChengGang released more versions of MaxDEA one after another, including MaxDEA Basic, MaxDEA Pro, and MaxDEA Ultra. MaxDEA Ultra is an optimization of MaxDEA Basic and MaxDEA Pro. MaxDEA Ultra contains all the functions of MaxDEA Basic and MaxDEA Pro, and the measurable DEA models included in this version are more comprehensive, faster, and support multi-core CPU parallel computing; so, this paper uses MaxDEA 8 Ultra software for measurement [59]. In this paper, we use the super-efficient MinDS model to measure the environmental performance values of the electric power industry in 30 provinces, autonomous regions, and municipalities under the direct control of the central government in China, and the results are shown in Table 3. The following is an analysis of the time-series characteristics of the environmental performance of China’s electric power industry from two perspectives: national and provincial (including autonomous regions and municipalities under the direct control of the central government), respectively.

#### 4.1.2. Trends in the Environmental Performance of the National Power Industry

**Figure 1:**
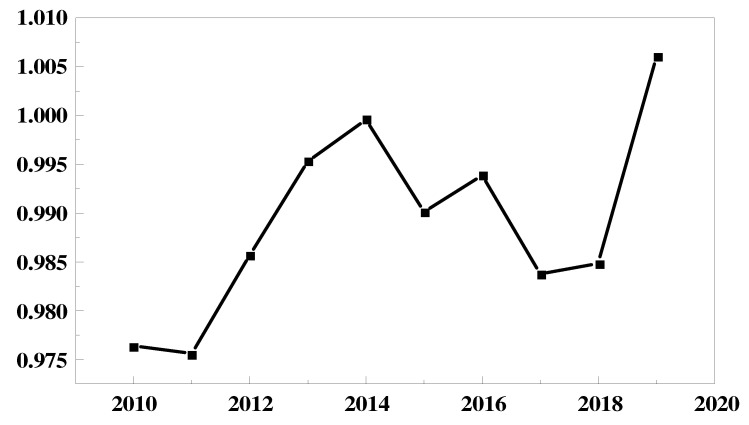
Trend of environmental performance of China’s power industry from the year 2010 to 2019.

From Figure 1, we can see that the overall environmental performance of China’s electric power industry from 2010 to 2019 shows an inverted U-shaped and then a U-shaped trend. Measuring the level of each year by the average level of each region, we can see that the environmental performance of the electric power industry rose from 0.98 in 2010 to 1 in 2014, with an increase of 2%. From 2014 to 2017, the environmental performance of the electric power industry showed a symmetrical decline. Starting in 2017, the environmental efficiency value of the electric power industry showed a symmetrical rise again to reach its highest level in 2019.

**Figure 2:**
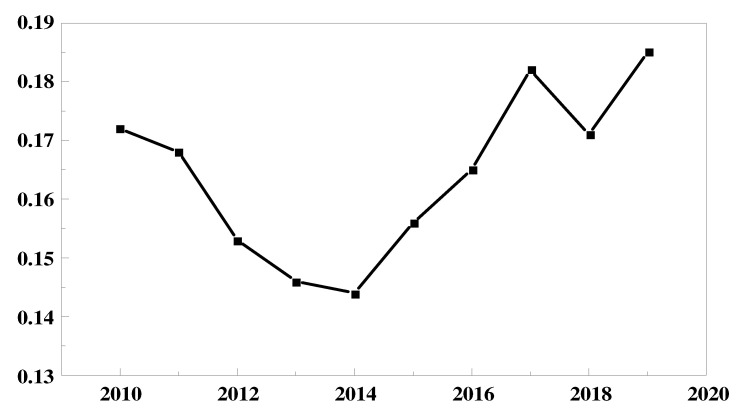
Trends in the standard deviation of environmental performance in China’s power industry from 2010 to 2019.

To reflect on the degree of variation in the environmental performance of the power industry in each region, we plotted the standard deviation of the environmental performance of the power industry in each region from 2010 to 2019 in Figure 2. From Figure 2, we can see that the regions approximate a symmetrical state of convergent development followed by divergent development, with 2014 as the structural change point. Among them, the discrete level of environmental performance of the electric power industry from 2010 to 2014 develops from 0.17 at a rate of 6% toward convergence to 0.14 in 2014. The same rate shows a change in the opposite direction from 2014 to 2019.

We believe that the possible reason for the above results is that, in 2011, the State Council issued the Opinions of the State Council on Strengthening Environmental Protection Priorities and the Notice of the State Council on the Issuance of the National Environmental Protection 12th Five-Year Plan, which proposed measures to control the total emissions of sulfur dioxide and nitrogen oxides in the power industry. The State Council’s “12th Five-Year Plan” notice proposed to control the total emissions of sulfur dioxide and nitrogen oxide in the power industry. Since 2011, power companies in all regions have raised their awareness of environmental protection and environmental pollution emissions in the power industry have been decreasing year by year; thus, environmental performance has begun to increase. Since in the initial stage, the sensitivity to environmental regulation was higher in regions with small power scales than in regions with large power scales, this led to the gradual reduction of individual differences between regions and the rate of reduction slowed down. Starting from 2014, the State Council issued the “Guidance on Further Promoting the Pilot Program of Paid Use and Trading of Emission Rights”, which proposed the establishment of a paid use system of emission rights. According to Wang and Liu [64], it is known that the collection of emission fees has a single threshold effect on the environmental performance of the power industry, i.e., the emission fees have a “cost effect” and “crowding out effect” on the power industry in the early stage of the system’s implementation. The “cost effect” and the “crowding out effect” are the same. The existence of the “cost effect” and “crowding out effect” has led to a lack of momentum in the power industry in regions with small electricity consumption and a weakened enthusiasm for energy conservation and emission reduction, while regions with large electricity consumption have continued to develop steadily based on social responsibility, leading to a gradual increase in individual differences between regions. The environmental performance of the power industry has declined in the short term; in 2016, the state issued the Opinions on the Implementation of a New Round of Rural Power Grid Renovation and Upgrading Project during the Thirteenth Five-Year Plan, proposing the goal of full coverage of power supply services in rural areas nationwide. Due to the expansion of the scale of electricity and the constraints of the natural conditions in rural areas, the investment in power supply equipment was large and costly, resulting in a decline in environmental performance. Since 2017, with the country’s emphasis on ecological environmental protection, the power industry has continued to develop and mature and the power structure has been optimized, resulting in an increase in environmental performance again.

#### 4.1.3. Trends in Environmental Performance of Electric Power Industry by Region

Considering the change and ranking of the environmental performance of the electric power industry from 2010 to 2019 in each region, we roughly classify the trend of change in the environmental performance of the electric power industry into three types, including rising, falling, and smoothness. The regions and characteristics included in each type are as follows.

(1) Rising type. This includes relatively economically developed areas such as Beijing, Hebei, Shanghai, Jiangsu, Fujian, Shandong, Sichuan, Yunnan, Jiangxi, Liaoning, Xinjiang, and Hainan. This is basically consistent with the conclusion reached by Peng [19]. The better development of environmental performance in the power industry in upwardly mobile regions is related both to the economically developed regions with a strong R&D investment base and to national policies. Taking Shanghai and Sichuan as examples, it can be seen from Table 3 that Shanghai’s electricity environmental performance has steadily increased from 1.18 in 2010 to 1.65 in 2019, showing an increase of 40%, and its change is also ranked first among all regions, which shows that Shanghai’s electricity industry environmental performance has risen the most. As the largest economic center in China, Shanghai follows the call of national policies, actively undertakes social responsibility, actively carries out scientific and technological innovation, continuously increases investment in research and development, establishes a coal consumption monitoring system, and increases the promotion of new technologies and equipment for energy conservation and emission reduction. Sichuan’s power environmental performance has steadily increased from 0.87 to 1.05, showing an increase of 20.35%, ranking in the top 10 in terms of change. Sichuan is mainly a hydroelectric power generator, and the country’s “west-to-east” strategy has created favorable conditions for its hydropower development. Due to the high cost of hydropower construction, the superiority of hydropower cannot be fully reflected in the early stages of construction. As hydropower projects are gradually put into use, the cost of hydropower is reduced; its advantages are thus highlighted. This, coupled with the expansion of the hydropower outbound market in Sichuan, has promoted the further development and utilization of hydropower. As a result, the environmental performance of Sichuan’s power industry shows an ever-increasing trend.

(2) Declining type. This includes Qinghai, Shanxi, Guizhou, Zhejiang, and Hubei. This is basically the same as the conclusion of the study by Guo et al. [23]. The declining regions are mainly in the central and western provinces. The declining environmental performance of the power industry in these regions is due to both their own resource endowment and industrial structure, as well as institutional reasons. Take Shanxi and Guizhou as examples. As can be seen from Table 3, the environmental performance of the power industry in Shanxi Province has declined by 15.21% from 2010 to 2019. The main reason is that Shanxi province’s power grid is “outward-oriented, transmission-oriented, and scale-oriented”, while it is currently facing a large surplus of domestic energy. Due to the insufficient capacity of cross-provincial and cross-regional transmission channels, it is not possible to meet the demand for outward transmission; in addition, since the second half of 2016, coal prices have continued to rise and downstream power generation enterprises are struggling to operate with a loss of up to 80%, resulting in a year-on-year decline in the environmental performance of Shanxi’s power industry. The environmental performance of electricity in Guizhou declined from 1.025 to 0.915, showing a decline of 10.78%. Guizhou’s economic development is relatively backward, and the conflict between supply and demand is prominent. A series of problems such as the lagging reform of the power investment system and the tariff management system have led to a lower level of environmental performance for its power industry.

(3) Smooth type. This includes Gansu, Guangxi, Hunan, Jilin, Shaanxi, Henan, Tianjin, Anhui, Heilongjiang, Inner Mongolia, Chongqing, Ningxia, and Guangdong. This is basically the same as the findings of Peng [19] and Guo et al. [23]. These regions are the regions that are in the bottom ten of the ranking of environmental performance changes in the electric power industry, and all of them exhibit small changes. We find that the smooth-type regions are mainly central and western provinces. The possible reason is that most of the smooth-type regions are still dominated by thermal power generation, which consumes mainly coal and therefore exerts great pressure on the environment. Although these regions have started to pay attention to energy conservation and emissions reduction in recent years and make efforts to develop clean energy, the withdrawal from the huge coal power system is bound to lead to high sunk costs and transition costs. At present, the reform of the thermal power generation industry is in a period of hard work, and the efficiency of reform has not yet significantly exceeded the efficiency of output, thus restricting the improvement of the level of environmental performance of the power industry.

#### 4.1.4. Spatial Characteristics of Environmental Performance in the Power Industry

The natural interruption point grading method is a map grading algorithm proposed by Chen et al. [65]. This is a method of identifying the classification intervals based on the natural groupings inherent in the data and grouping similar values most appropriately so that the differences between the classes are maximized. This method uses the idea of clustering, but while clustering does not focus on the number and range of elements in each class, the natural interruption point method tries to ensure that the range and number of elements between each group are as similar as possible. Therefore, we used the natural interruption point hierarchy method to study the spatial characteristics of environmental performance in the electric power industry. According to the average level of environmental performance of the power industry in each region from 2010 to 2019, we classified each region into four levels—ultra-high level, high level, medium level, and low-level regions. The results of the classification are shown in Table 4.

(1) Ultra-high-level regions. From Table 4, we can see that the ultra-high-level regions mainly include two categories: one is the more developed eastern region, and the other is the region with the advantage of PV power generation. This is roughly the same as the findings of Fan and Yuan [66], Luo [67], Jiang [68], and others. However, the division of high-level areas in this paper is more detailed, and this paper divides high-level areas into ultra-high-level and high-level regions. This detailed division is more conducive to discovering the differences in the environmental performance of the electric power industry between different high-level regions and is more conducive to discovering the advantages of high environmental performance in the electric power industry. We believe that the main reasons for these results are that Beijing and Shanghai are the political and economic centers of China, respectively, and are the main importers of electricity from the west to the east, thus generating less pollutants in their own power generation. In addition, these two cities have a high level of economic development and civilization and pay more attention to investment in ecological and environmental management; so, the environmental performance of the power industry is at a very high level; Jiangsu Province, with the advantage of containing the Yangtze River Delta, has achieved rapid economic development, and although the overall electricity consumption is high, it is mainly dominated by strategic emerging industries and manufacturing industries with high technology content, and the output efficiency per unit of electricity consumption is much higher than average. The environmental performance of the power industry is therefore high. Hainan Province, with a small population and a lack of industrial clusters, consumes less electricity and therefore emits less environmental pollution and has a higher level of environmental performance. Qinghai Province, relying on its rich natural resources, mainly produces hydropower and photovoltaic power, supplemented by coal and wind power, and has the world’s largest concentration of grid-connected photovoltaic power, making it one of the provinces with a higher environmental performance in the power industry.

(2) High-level regions. As we can see from Table 4, there are four main categories of high-level region. The first category is regions with complete power generation structures, the second category is economically developed regions, the third category is regions that pay attention to the control of carbon emissions in the power industry, and the fourth category is the region with clean energy generation. Inner Mongolia is the leading source of electricity in China, with a perfect power structure involving thermal power generation, wind power generation, and solar power generation. It has ranked first in the country in terms of outgoing power for many years, and a large amount of outgoing power transfers part of the environmental pollution to the power input province, reducing the environmental pollution emissions in the power output province; so, the expected output quantity of electricity in Inner Mongolia is more and the non-expected output is less, which greatly contributes to the environmental performance of the power industry. Tianjin, Fujian, and Guangdong all belong to regions with high levels of economic development and high investment in environmental management. Additionally, they mainly house light industry, so the power pollution emissions are lower. They therefore have a high-level industry environmental performance. Although the Anhui province power industry has high carbon emissions, the development of the power industry carbon emission reduction schedule has led to favorable power industry carbon emissions reductions (for example, the Emission Standards for Air Pollutants from Thermal Power Plants and the List of Key Emission Units in the Power Generation Industry). Yunnan, Hubei, and Sichuan rely on abundant water resources and mainly use hydroelectric power generation. They are thus clean energy provinces with less environmental pollution emissions and therefore have higher environmental performance in the power industry. Although Ningxia is mainly a thermal power generation province, it has a photovoltaic power generation industrial park, which contributes to the “west–east power transmission”. Therefore, the environmental performance of Ningxia’s electric power industry is relatively high.

(3) Medium-level regions. From Table 4, we can see that these regions are generally characterized by a high proportion of high-energy-consuming industries and low value-added industries. For example, regions such as Shanxi, Shaanxi, Chongqing, and Shandong have a huge share of thermal power generation and a negligible amount of clean energy generation. In particular, Shaanxi and Shanxi, as resource-based provinces, are dominated by high-energy industries and thus have huge electricity consumption. However, at the same time, these industries are not high-value-added industries; so, the overall return rate is relatively low. This eventually causes a mismatch between electricity consumption and economic growth, leaving the environmental performance of the power industry at a medium level.

(4) Low-level regions. As shown in Table 4, the low-level regions are mainly the central and western provinces, which are generally characterized by the predominance of thermal power generation and a low level of economic development. The reason for the different results may be related to the time span of the study. In recent years, some western regions have relied on the advantages of natural conditions to develop clean energy, and thus the power sector has gradually moved away from low-level environmental performance. Heilongjiang, Jilin, and Liaoning, as old industrial bases in Northeast China, have been relying on crude heavy industry, resources, and energy as pillar industries, with high power consumption but insufficient power capacity. Moreover, the three northeastern provinces mainly focus on thermal power generation while the power environment is seriously polluted and the economic development level is relatively backward, so the environmental performance is at a low level. Henan province also mainly relies on thermal power generation, and the power industry has more labor input, which leads to low environmental performance. Gansu and Xinjiang are in the northwest region, and their economic development level is relatively backward. Additionally, they rely mainly on thermal power generation, which makes the environmental pollution worse. This has led to more environmental pollution and lower environmental performance.

### 4.2. Redundancy Analysis of the Environmental Performance of China’s Power Industry

In the super-efficient MinDS model, the slack variable represents an optimization quantity, which indicates the “distance” of the DMU from the effective frontier, which reflects the input redundancy and output deficiency of the DMU. In order to measure the relative redundancy level of each input–output indicator, we define the redundancy index. The redundancy degree is expressed as the ratio of the absolute amount of the slack scalar of each indicator to the original value of the input or output indicator. In this study, we first screened out the regions with efficiency values less than 1 from 2010 to 2019; i.e., the inefficient regions that needed improvement. Then, the redundancy of each indicator in these regions was calculated. Using the redundancy degrees of these indicators, it is possible to analyze both the gap between each indicator and the optimal level of environmental performance of the power industry nationwide and in each region, as well as their trends. It provides a clear guiding direction for the enhancement and improvement of the environmental performance of the power industry nationwide and in each region.

#### 4.2.1. Analysis of the Redundancy Degree of the Environmental Performance of the National Power Industry

**Figure 3:**
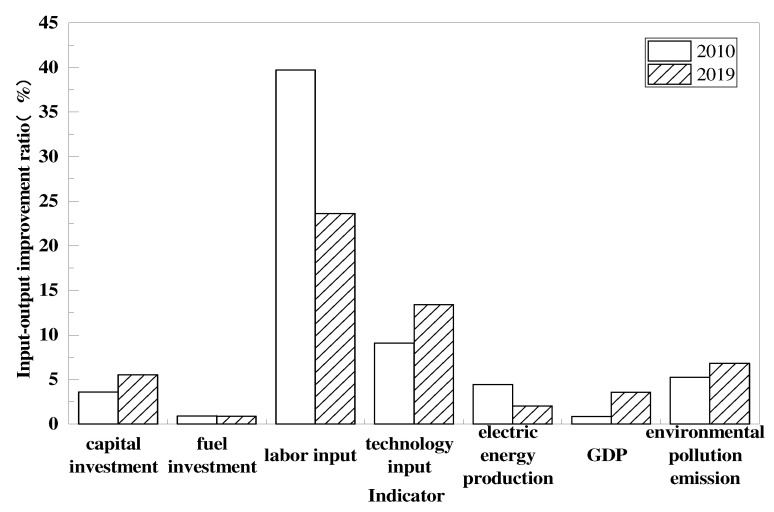
Percentage improvement of each indicator in 2010 and 2019.

We used the average of each indicator in the inefficient regions from 2010 to 2019 to represent the redundancy of each input–output indicator of environmental performance of the national power industry from 2010 to 2019. We use histograms to represent the redundancy of each indicator of the environmental performance of the electric power industry in China in 2010 and 2019, as shown in Figure 3.

From Figure 3, we can see that, as a whole, the redundancy of input–output indicators in China’s power industry is from high to low in the order of labor input, technology input, environmental pollution emission, capital input, power generation, gross regional product, and fuel input. Compared with 2010, the redundancy of fuel input, labor input, and power generation in the electric power industry decreased in 2019. Of these, labor input redundancy declined the most, by 16%, which matches the reality of the gradual shift from a demographic dividend to a technological dividend in our economic growth. The redundancy of capital input and technology input and the shortage of output indicators, such as regional GDP and environmental pollution emissions, have all increased. Among them, the redundancy of technology input has risen the most, which is more related to the excessive consumption of plant electricity rate and line loss rate of electric power enterprises in China. The increase in the deficiency by regional GDP indicates that electricity, as one of the factors of production, is declining in its contribution to economic development. We argue that the reason for this is that the pollution generated by the electric power industry affects the health level and thus the labor productivity. Therefore, the increase in the contribution of electricity as a factor of production to economic growth is offset by the damage to economic growth from pollution in the electricity sector.

#### 4.2.2. Analysis of the Redundancy of the Environmental Performance of the Power Industry in Each Province

**Figure 4:**
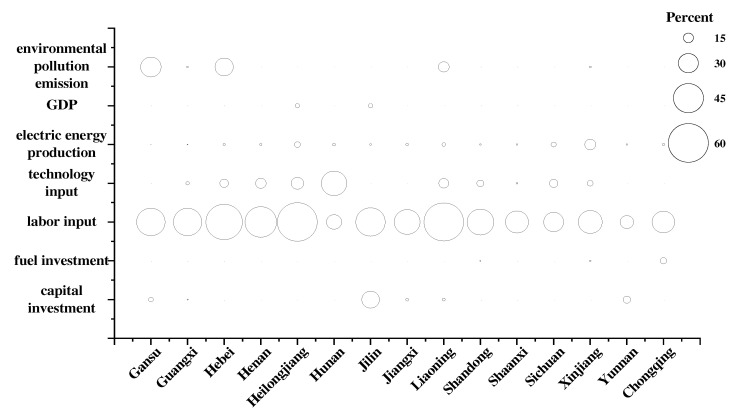
Percentage of improvement in inefficient provinces in the year 2010.

**Figure 5:**
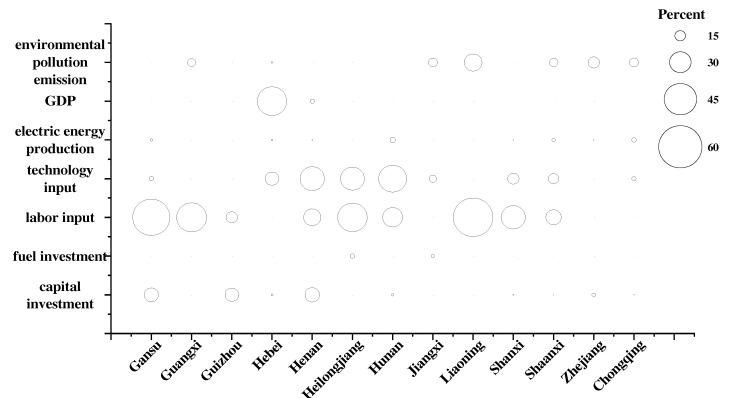
Percentage of improvement in inefficient provinces in the year 2019.

In order to explore the gap between each indicator and the optimal level of environmental performance in the power industry in each region and the trend of change, we drew bubble diagrams, as shown in Figure 4 and Figure 5. In Figure 4 and Figure 5, the area of the bubble indicates the redundancy of the indicator, and the larger the area the greater the redundancy of the indicator in the region and the greater the proportion of improvement needed. From the results, we can find that from 2010 to 2019, Guizhou, Shanxi, and Zhejiang changed from efficient provinces to inefficient provinces, while Jilin, Shandong, Sichuan, Xinjiang, and Yunnan changed from inefficient provinces to efficient provinces. In terms of indicator redundancy, the majority of indicators have improved in varying degrees from 2010 to 2019, with the greatest improvements being seen in labor input redundancy. This also confirms the phenomenon mentioned earlier in this paper. With the disappearance of China’s demographic dividend and the improvement of labor productivity, the waste of labor resources in each region has been significantly reduced. From the current situation, there is redundancy of capital input in Gansu, Guizhou, and Henan, and these regions should be limited in their installed capacity. There is redundancy of technical input in Hebei, Henan, Heilongjiang, Hunan, Jiangxi, Shanxi, and Shaanxi, and these regions should make efforts to reduce the consumption of plant electricity rate and line loss rate. There is excessive undesired output of environmental pollution emission in Guangxi, Jiangxi, Liaoning, Shaanxi, Zhejiang, and Chongqing; therefore, these regions should redouble their efforts to promote the development of advanced power generation technologies that have high efficiencies, low emissions, and multiple cycles.

## 5. Conclusions and Insights

Based on previous studies, this paper constructs an environmental performance evaluation index system based on the electric power industry as an example, by which the environmental performance level of key polluting industries in China is evaluated, the spatial and temporal characteristics of the environmental performance level of each region in China are explored, the gap between the current environmental performance level and the optimal level is quantitatively analyzed, and the path to improve the environmental performance level of key polluting industries in China is proposed. The main contributions of this paper are threefold. First, we incorporate technological inputs into the environmental performance evaluation index system of the electric power industry, which can better reflect the real inputs of the electric power industry and measure the results more accurately. Second, we adopt the MinDS model for measuring the environmental performance level, which can quantitatively analyze the gap between each indicator and the optimal level. Third, we propose a redundancy index, which can be used to compare the redundancy of each indicator and then judge the main efficiency levels of different factors. The main conclusions drawn in this paper are as follows.

(1) The overall environmental performance of China’s electric power industry shows the characteristics of an inverted U-shaped first and then a U-shaped trend. The environmental performance of the electric power industry in each region presents an asymmetrical state with 2014 as the structural change point, with convergence development coming first followed by differentiation development.

(2) The trend of environmental performance changes in the electric power industry in each region can be roughly divided into three categories: rising, falling, and stable. The rising type includes Beijing, Hebei, Shanghai, Jiangsu, Fujian, Shandong, Sichuan, Yunnan, Jiangxi, Liaoning, Xinjiang, and Hainan. The falling type mainly includes Qinghai, Shanxi, Guizhou, Zhejiang, and Hubei. The stable type mainly includes Gansu, Guangxi, Hunan, Jilin, Shaanxi, Henan, Tianjin, Anhui, Heilongjiang, Inner Mongolia, Chongqing, Ningxia, and Guangdong.

(3) The spatial clustering effect of the environmental performance of the power industry in each region is divided into four types: ultra-high level, high level, medium level, and low level. The ultra-high-level regions are Beijing, Shanghai, Jiangsu, Hainan, and Qinghai. The high-level regions are Inner Mongolia, Tianjin, Anhui, Fujian, Guangdong, Yunnan, Hubei, Sichuan, and Ningxia The medium-level regions are Shanxi, Shaanxi, Chongqing, Guizhou, Jiangxi, Zhejiang, and Shandong. The low-level regions are Heilongjiang, Jilin, Liaoning, Hebei, Henan, Hunan, Gansu, Xinjiang, and Guangxi.

(4) The input–output indicators of environmental performance in China’s power industry have different degrees of redundancy. The redundancy of labor input is the largest, followed by capital input, and finally technology input, power generation, regional GDP, and fuel input. There is redundancy in capital inputs in Gansu, Guizhou, and Henan; redundancy in technology inputs in Hebei, Henan, Heilongjiang, Hunan, Jiangxi, Shanxi, and Shaanxi; and excessive non-desired outputs of environmental pollution emissions in Guangxi, Jiangxi, Liaoning, Shaanxi, Zhejiang, and Chongqing.

(5) The inefficient provinces are not invariant across regions. During the analysis period, Guizhou, Shanxi, and Zhejiang moved from efficient provinces to the ranks of inefficient provinces, and Jilin, Shandong, Sichuan, Xinjiang, and Yunnan moved from inefficient provinces to the ranks of efficient provinces; the redundancy degree of most indicators in each inefficient province has improved to different degrees, among which the labor input redundancy has improved the most.

This paper argues that efforts should be made in the following aspects to promote the improvement of the environmental performance level of key polluting industries in China.

(1) Improve the supervision and inspection mechanism of the implementation process of pollution control in key polluting industries. From the previous analysis, it can be seen that the release of environmental pollution control-related documents can greatly enhance the environmental awareness of environmental pollution subjects, but the results of the implementation process vary greatly from region to region. The implementation process should be concretized and involve the development of emission reduction standards, regular publication of the list of exceedances, proposed corrective measures, and tracking implementation. This is the only way to fundamentally improve the environmental performance of key polluting industries.

(2) Each region should adopt differentiated strategies to improve the environmental performance of key polluting industries according to local conditions. Regions with a high level of economic development should make full use of their economic base to develop carbon emission reduction technologies and actively export them to maximize the technology spillover effect. For western regions with inherent advantages, they should make full use of their own advantages to build photovoltaic power parks to serve the national strategy of “west–east power transmission”. For areas with mainly thermal power generation, it is difficult to realize the transformation of the power industry structure in a short period of time; so, the production efficiency of the original power generation method should be improved. In short, the whole country should play to the strengths of each region to complement each other’s weaknesses and optimize the environmental performance of key polluting industries as a whole.

(3) Optimize the combination of labor, capital, and technology input factors and improve the efficiency of factor inputs. Labor, capital, and technology inputs are important factors that limit the level of environmental performance of key polluting industries in China and in each region. Today, when the demographic dividend no longer exists, improving labor efficiency is a necessity. The excessive redundancy of labor should be reduced by streamlining personnel, improving the intellectual quality and skill quality of labor, and establishing an effective wage-restraint mechanism. Key polluting industries tend to invest more, and the excessive investment of capital is a serious waste of resources, which should be eliminated by replacing outdated equipment, accurate measurements, and updating as needed. The excessive investment of technology is mainly caused by high plant electricity rates and excessive consumption of line loss rates, which should be vigorously targeted via research on and development of low energy consumption wind and smoke systems, desulfurization systems and powder making systems, power grid transformations, and sound line loss management systems. The inputs of labor, capital, and technology cannot be considered in isolation; only by optimizing the quantity and quality of labor, capital, and technology can we fundamentally improve the environmental performance of key polluting industries in China and its regions.

This paper analyzes the environmental performance, spatial and temporal characteristics, and optimization paths of key polluting industries, represented here by the electric power industry, using the super-efficient MinDS model. In the future, we can use the super-efficient MinDS–Malmquist combination model to further analyze the changes in environmental technology in the electric power industry and reveal the sources of environmental performance changes. According to our analysis, the environmental performance of China’s electric power industry shows a rising–declining–rising trend, and the standard deviation shows a declining–rising trend, indicating that the individual differences between different regions first decreased and then expanded, which may be related to the scale of the electric power industry and environmental regulation. There may be a threshold effect, which should further be explored in the future.

## Figures and Tables

**Table 1:** Construction of the environmental performance index system in the power industry.

Category	Primary Indicator	Secondary Indicators	Unit
Input indicators	Capital investment	Installed capacity	million kilowatts
	Fuel input	Consumption of standard coal for power generation	million tons
	Labor input	Number of workers	million people
	Technical input	The electricity consumption rate of power plants	%
		Line Loss Rate	%
		Length of power transmission lines	Kilometers
Output indicators	normal product	Electricity generation	Kilowatt-hour
		GDP	Billion Yuan
	Environmental Pollution Emissions	CO_2_ emissions	million tons
		SO_2_ emissions	million tons
		Nitrogen oxides	million tons

**Table 2:** Descriptive statistics of environmental performance indicator variables in the power industry.

Statistical Quantities	Average	Standard Deviation	Minimum	Maximum	Median
Installed power generation capacity	4713	3079	66	14,044	4196.46
Standard coal consumption	5594	3796	66.42	17,043	4805.236
Employment	11.94	6.701	0.740	32.18	11.43
Electricity consumption rate of power plants	4.915	1.732	0.500	8.400	5.15
Line loss rate	6.443	1.926	2.230	13.80	6.4
Length of transmission line	52,394	26,904	6507	118,665	55,994.5
Electricity generation	1872	1283	21.02	5897	1614.7
GDP	20,272	17,314	512.9	90,788	15,442
CO_2_ emissions	18,317	13,758	199.4	66,759	14,376.74
SO_2_ emissions	551.2	414.0	6	2009	432.6
Nitrogen oxides	275.6	207.0	3	1004	216.3

**Table 3:** Environmental performance values of the power industry by region from the year 2010 to 2019.

DMU	2010	2011	2012	2013	2014	2015	2016	2017	2018	2019	Standard Deviation
Anhui	1.032	1.054	1.041	1.027	1.028	1.017	1.009	1.011	1.017	1.010	0.014
Beijing	1.351	1.474	1.430	1.391	1.376	1.401	1.445	1.505	1.456	1.399	0.045
Fujian	1.001	1.006	1.005	1.004	0.991	0.998	0.999	1.008	1.019	1.029	0.010
Gansu	0.796	0.791	0.851	0.905	0.859	0.735	0.719	0.726	0.773	0.800	0.058
Guangdong	1.058	1.069	1.052	1.044	1.040	1.054	1.052	1.041	1.060	1.040	0.009
Guangxi	0.867	0.854	0.860	0.816	0.883	0.835	0.861	0.787	0.901	0.865	0.031
Guizhou	1.025	0.848	1.015	1.003	1.101	0.832	0.712	0.899	0.901	0.915	0.108
Hainan	1.159	1.071	1.133	1.140	1.166	1.168	1.163	1.201	1.166	1.235	0.041
Hebei	0.757	0.732	0.764	0.794	0.833	0.839	0.826	0.816	0.852	0.824	0.038
Henan	0.836	0.864	0.860	0.893	0.870	0.854	0.864	0.842	0.848	0.789	0.026
Heilongjiang	0.769	0.778	0.768	0.759	0.786	0.800	0.798	0.771	0.779	0.802	0.014
Hubei	1.090	1.086	1.106	1.040	1.053	1.042	1.032	1.028	1.034	1.021	0.028
Hunan	0.838	0.872	0.858	0.861	0.826	0.807	1.001	1.003	0.785	0.809	0.073
Jilin	0.802	0.824	0.822	0.807	0.816	1.002	1.000	0.742	0.799	1.011	0.095
Jiangsu	1.123	1.123	1.154	1.198	1.195	1.150	1.209	1.252	1.242	1.191	0.043
Jiangxi	0.884	0.897	0.847	0.862	0.861	0.855	0.943	0.918	0.913	0.924	0.032
Liaoning	0.754	0.774	0.774	0.813	0.849	0.824	0.814	0.814	0.822	0.800	0.027
Inner Mongolia	1.043	1.061	1.055	1.072	1.076	1.068	1.041	1.063	1.065	1.081	0.012
Ningxia	1.013	1.065	1.051	1.069	1.076	1.054	1.070	1.101	1.086	1.074	0.022
Qinghai	1.513	1.363	1.266	1.221	1.206	1.192	1.135	1.079	1.108	1.135	0.125
Shandong	0.864	0.876	0.895	0.937	0.887	1.053	1.042	1.020	1.045	1.038	0.076
Shanxi	1.030	1.011	1.014	1.006	0.969	0.843	1.001	0.826	0.883	0.873	0.076
Shaanxi	0.903	0.927	1.018	1.044	1.048	1.046	1.031	1.021	0.871	0.864	0.073
Shanghai	1.177	1.209	1.211	1.266	1.290	1.304	1.307	1.325	1.364	1.652	0.127
Sichuan	0.873	0.917	0.919	1.018	1.042	1.056	1.052	1.059	1.047	1.051	0.068
Tianjin	1.052	1.045	1.039	1.045	1.015	1.034	1.025	1.017	1.028	1.013	0.013
Xinjiang	0.833	0.807	0.849	0.859	0.917	0.804	0.696	0.664	0.711	1.005	0.099
Yunnan	0.918	0.917	0.941	1.007	1.044	1.063	1.087	1.084	1.086	1.070	0.067
Zhejiang	1.024	1.014	1.017	1.005	0.934	1.019	0.920	0.923	0.924	0.936	0.045
Chongqing	0.883	0.914	0.943	0.948	0.950	0.945	0.956	0.952	0.945	0.927	0.021
Standard deviation	0.172	0.168	0.153	0.146	0.144	0.156	0.165	0.182	0.171	0.185	0.165

**Table 4:** Spatial clustering effect of environmental performance by natural interruption point grading method.

Type	Region
Ultra-high level	Beijing, Shanghai, Jiangsu, Hainan, Qinghai
High level	Inner Mongolia, Tianjin, Anhui, Fujian, Guangdong, Yunnan, Hubei, Sichuan, Ningxia
Medium level	Shanxi, Shaanxi, Chongqing, Guizhou, Jiangxi, Zhejiang, Shandong
Low level	Heilongjiang, Jilin, Liaoning, Hebei, Henan, Hunan, Gansu, Xinjiang, Guangxi

## Data Availability

Not applicable.
